# Digital Health Interventions for Informal Family Caregivers of People With First-Episode Psychosis: Systematic Review on User Experience and Effectiveness

**DOI:** 10.2196/63743

**Published:** 2024-11-28

**Authors:** Pauline Sarah Münchenberg, Dinara Yessimova, Dimitra Panteli, Tobias Kurth

**Affiliations:** 1 Institute of Public Health Charité – Universitätsmedizin Berlin Berlin Germany; 2 Department of Health Care Management Technische Universität Berlin Berlin Germany; 3 European Observatory on Health Systems and Policies Brussels Belgium

**Keywords:** first-episode psychosis, early psychosis, digital health interventions, telepsychiatry, informal caregivers, family caregivers, telehealth, severe mental disorders, psychosis, digital intervention, digital health, mental health, psychoeducation, mobile phone

## Abstract

**Background:**

First-episode psychosis (FEP) imposes a substantial burden not only on the individual affected but also on their families. Given that FEP usually occurs during adolescence, families overtake a big part of informal care. Early family interventions, especially psychoeducation, are crucial for informal family caregivers to best support the recovery of their loved one with FEP and to reduce the risk of a psychotic relapse as much as possible, but also to avoid chronic stress within the family due to the burden of care. Digital health interventions offer the possibility to access help quicker, use less resources, and improve informal family caregiver outcomes, for example, by reducing stress and improving caregiver quality of life.

**Objective:**

This study aimed to systematically identify studies on digital health interventions for informal family caregivers of people with FEP and to describe and synthesize the available literature on user experience, as well as the effectiveness of such digital applications on the clinical outcomes, consisting of (1) perceived caregiver stress, (2) expressed emotion, and (3) parental self-efficacy.

**Methods:**

A systematic search was carried out across 4 electronic databases. In addition, reference lists of relevant studies were hand-searched. This review aimed to include only primary studies on informal family caregivers, who had to care for a person with FEP between 15 years and 40 years of age and a diagnosis of FEP with onset of observed symptoms within the past 5 years. All types of digital interventions were included. This systematic review is aligned with the PRISMA (Preferred Reporting Items for Systematic Reviews and Meta-Analysis) 2020 guidelines.

**Results:**

The search identified 7 studies that reported on user experience or effectiveness of digital health interventions on perceived caregiver stress, expressed emotion, and parental self-efficacy, including 377 informal family FEP caregivers across trials. Digital health interventions–web-based, videoconferences, and mHealth–were well accepted and perceived as relevant, easy to use, and helpful by informal family FEP caregivers. Psychoeducational content was rated as the most important across studies. Perceived caregiver stress, expressed emotion, and parental self-efficacy improved in all studies that reported on these clinical outcomes.

**Conclusions:**

The results of this review suggest that digital health interventions aimed at informal family caregivers of individuals with FEP can improve relevant clinical outcomes, with participants reporting a positive user experience. However, for some interventions reviewed, specialized in-person family care outperformed the digital intervention and partially led to better results in perceived caregiver stress and parental self-efficacy. Therefore, while digital interventions present a promising approach to alleviate the burden of care and improve informal family FEP caregiver outcomes, more studies with well-powered experimental designs are needed to further investigate the effectiveness of such applications in this population.

**Trial Registration:**

PROSPERO CRD42024536715; https://tinyurl.com/bdd3u7v9

## Introduction

First-episode psychosis (FEP) usually occurs during adolescence with age of onset mostly between 15 and 25 years of age and imposes a substantial burden of disease on the individual affected, their families, and society [1-4]. Psychotic disorders describe a group of mental health conditions that present—among other possible symptoms—with delusions, hallucinations, disorganized speech, and grossly disorganized or catatonic behavior [5]. FEP often represents a time of crisis for people affected and their families and, given that many people with FEP do not seek professional help, there is a significant unmet public health need for care, especially in young people [6-9]. Recovery after FEP requires long-term treatment and often has a negative effect on the quality of life, social connections, education, employment, and independent functioning, not only for the individual with psychosis but also for their informal family caregivers and society [10].

Early interventions are crucial, as treatment benefits for people with psychotic disorders are much greater when the duration of untreated psychosis (DUP) is shorter as opposed to longer [11-13]. Most patients still live in their family home at the onset of psychosis and their families play a big part in providing informal care throughout their recovery process [14]. Family support throughout the course of the psychotic disorder is associated with a more efficient use of health care services, treatment adherence, and better health outcomes for the patient [15,16]. Further, a positive and warm family environment seems to be a protective factor in the improvement of functional outcomes of patients with FEP, while caregiver criticism and hostility (known as expressed emotion) are risk factors for a psychotic relapse [17-19]. However, given the high burden of care related to FEP, informal family caregivers are often under high stress, experience physical and psychological health problems, and have a reduced quality of life [20]. Further, the burden of care is often increased by negative illness beliefs, avoidant-focused coping styles, and family conflict, amongst other factors, as well as the fact that the majority of people with FEP will experience psychotic relapses throughout the course of psychosis [19,21].

To alleviate the burden of care and to equip informal family caregivers with appropriate coping mechanisms during the challenging time of an FEP diagnosis, family interventions such as psychoeducation, reducing negative aspects of expressed emotion, clinical guidance, and psychological support are of high importance [22]. Such interventions are especially effective in the early stages of FEP, just like interventions for the individual with FEP should commence as soon as possible after the onset of psychosis [18,23]. However, informal family caregivers often face an unmet need for education and skill training, effective coping strategies, awareness, and support [24]. Therefore, it is crucial to engage informal family caregivers in the right form of care early on to provide them with more flexibility when seeking help and to meet their needs, while supporting the recovery of their loved one and improving their health outcomes at the same time [17,25,26].

Family interventions can be delivered in person or online through digital health applications. The ability to provide medical care online by using telecommunication and IT is rapidly advancing and increasingly used in today’s health care systems [27,28]. Especially in health care areas such as psychiatry, where patients and their caregivers are often in need of a specialist or may be unable to travel to a doctor’s office, digital health is well suited, as it resolves barriers such as shortage of specialized personnel in rural areas or the patient’s inability to leave the house [29]. Some of the common barriers informal family caregivers of people with FEP face include accessing family support, help to develop coping strategies, support from social networks, and education on psychosis [30]. These barriers are often related to high costs of service, travel distances, and lack of resources such as transportation. Further, informal FEP family caregivers often have difficulties navigating the mental health system, resulting not only in a potentially longer DUP as family members are often the ones initiating treatment for the individual with FEP, but also in higher caregiver distress [30,31]. Stigmatization toward psychosis remains another reason why accessing mental health services is often delayed and why informal family caregivers of individuals with FEP feel isolated and judged by their social network [31,32]. In recent years, there has been a bigger focus on delivering digital-based interventions to informal family caregivers of people with mental illnesses [33]. Digital health interventions have the significant potential to improve health and health care delivery by being scalable, affordable, and sustainable tools, which are often more reachable by people in need compared with traditional in-person interventions, while offering the possibility of data collection to advance research and knowledge [30,34-36]. Further, such interventions can provide a sense of privacy as they are delivered digitally and may reduce problems surrounding stigmatization while increasing access to mental health services [30]. Preliminary evidence suggests that digital health interventions may offer an effective way to support informal family caregivers, leading to improvements in caregiver outcomes and high caregiver satisfaction, while enabling them to access remote support and education more easily, saving time and resources [37-40]. However, robust studies have only been published in recent years and challenges related to implementing digital health technologies in real-world settings remain, such as data safety, regulatory hurdles, reimbursement of telepsychiatry services, and, crucially, engagement and digital literacy [34].

The goal of this systematic review is to investigate informal family FEP caregivers’ user experience with digital health solutions, as well as the effectiveness of such interventions on perceived caregiver stress, expressed emotion, and parental self-efficacy. These outcomes are especially relevant in the recovery process of the individual with FEP, given that they play a central role in preventing relapses. This study will help to understand how digital interventions may improve informal family caregivers’ quality of life, functioning, and mental well-being and may inform future health strategies related to treatment inclusion and support of informal family caregivers of people with FEP. Further, given the promise of digital health technologies for reducing costs and barriers to access, this review may help inform future policy and practice about designing and providing such options for informal family caregivers of people with FEP, while addressing common challenges related to the implementation of digital health interventions.

## Methods

### Trial Registration

The research protocol for this study was registered on PROSPERO (CRD42024536715) before performing the systematic review.

### Selection Criteria

The research questions were framed based on the PICO model (Population Intervention Comparison Outcome) [41], refer to Textbox 1.

The goals of this review are to (1) investigate informal family FEP caregivers’ user experience with digital health interventions; (2) explore the effectiveness of digital health interventions on perceived caregiver stress, expressed emotion, and parental self-efficacy in informal family caregivers of people with FEP; and (3) provide recommendations for future policy and practice whilst being mindful of the limited evidence.

The inclusion and exclusion of this study are listed in Textbox 2.

**Research question based on the Population Intervention Comparison Outcome (PICO) model.**

(P) population:

- Informal family caregivers providing support for a loved one affected by first-episode psychosis

(I) intervention:

- Caregiver-focused digital health interventions

(C) comparison:

- Usual care (where applicable)

(O) outcomes:

- User experience with digital health interventions, or effectiveness of digital health interventions on perceived caregiver stress, expressed emotion, and parental self-efficacy (within the scope of this review, we defined parental self-efficacy as perceived self-efficacy, perception of competence, and (coping) confidence as a parent [42])

**Inclusion and exclusion criteria.**

**Inclusion criteria**

- Papers published in peer-reviewed journals and written in English (studies from all countries were eligible, but the language of publication was restricted to English).
- The study aimed to investigate informal family first-episode psychosis (FEP) caregivers’ user experience with digital health interventions, and improvement of the clinical outcomes of perceived stress, expressed emotion, and parental self-efficacy in this population.
- The study intervention had to be administered in a digital format (for example, through the internet or mobile app).
- Informal family caregivers had to care for a patient who was between 15 years and 40 years of age and had a diagnosis of FEP with onset of observed psychotic symptoms within the past 5 years.

**Exclusion criteria**

- Papers not published in peer-reviewed journals, literature reviews, and studies describing experimental protocols with no current results-other than study protocols, all study designs were eligible to include as many studies as possible, as long as the paper was written in English.
- Informal family caregivers in the study sample cared for patients who had comorbidities or a diagnosis of severe mental illness different than FEP, with onset earlier than within the past 5 years, and younger than 15 or older than 40 years of age. Although the age of onset of FEP is mostly between 15 years and 25 years, we only excluded people with late-onset psychosis (start of symptoms between 40 and 60 years of age) to include as many papers as possible in our search. Late-onset psychosis requires different clinical considerations and the prevalence of FEP is lower in older adults [1,43,44].

### Search Strategy

A systematic search of the international literature was carried out using 4 electronic databases (MEDLINE, PubMed, Embase, and PsycINFO) to identify relevant studies on the topic from database inception to March 1, 2024. Studies for review were identified following a keyword and MeSH (Medical Subject Headings) term search using the search terms, such as FEP, early psychotic disorder, informal caregivers, family caregivers, digital health, telemedicine, telepsychiatry, and telehealth. The search used the Boolean operators “OR” and “AND” to combine the keywords and MeSH terms effectively. The search equation used the title, abstract, and keyword fields. Bibliographical database searches were supplemented by hand-searching references of relevant studies and previous systematic reviews [39,45]. The search was carried out in English.

Search results were uploaded into Rayyan, a web and mobile app for systematic reviews, and duplicates were removed by performing a duplicate check with Rayyan [46]. A first round of title and abstract screening was followed by a full-text screening of eligible studies and data extraction of relevant information. This included data on (1) study characteristics (for example, authors, publication year, and sample size characteristics), (2) digital health intervention (for example, type and key components), (3) study outcomes on user experience with the digital intervention, and (4) study outcomes on perceived stress, expressed emotion, and parental self-efficacy. The extracted information was collected in a standardized table in Google Forms. Further, studies from the same institution were examined to make sure that individual studies provided new information, thus being separate studies and not one study with multiple publications to avoid duplication.

The screening and data extraction was carried out independently by 2 reviewers (PM and DY). Discrepancies were solved through discussion and by involving a third reviewer (DP) where needed, reaching a consensus on whether to include each paper and the extracted information. The search procedure followed the PRISMA (Preferred Reporting Items for Systematic Reviews and Meta-Analyses) 2020 guidelines. The PRISMA 2020 checklist is listed in Multimedia Appendix 1 [47]. The full search strategy, including a complete list of the keywords and MeSH terms, can be found in Multimedia Appendix 2.

### Risk of Bias and Methodological Quality

The risk of bias in randomized controlled trials (RCTs) was assessed using the Cochrane risk of bias 2 tool [48]. For nonrandomized studies, the ROBINS-I tool (Risk of Bias in Non-randomized Studies - of Interventions) [49] was chosen. Each study was given an overall rating of risk of bias (low risk; some concerns or moderate; or high risk).

The methodological quality of the included studies was evaluated using the Quality Assessment Tool for Quantitative Studies (QATO) [50]. Each study was assigned a global rating across eight assessment categories, including selection bias, study design, and confounding among other domains (strong=no weak ratings; moderate=one weak rating; or weak=2 or more weak ratings).

Risk of bias and methodological quality were rated independently by PM and DY for all papers and consensus was reached in case of discrepancies through discussion and by involving a third reviewer (DP) where needed.

## Results

### Study Selection and Data Extraction

**Figure 1:**
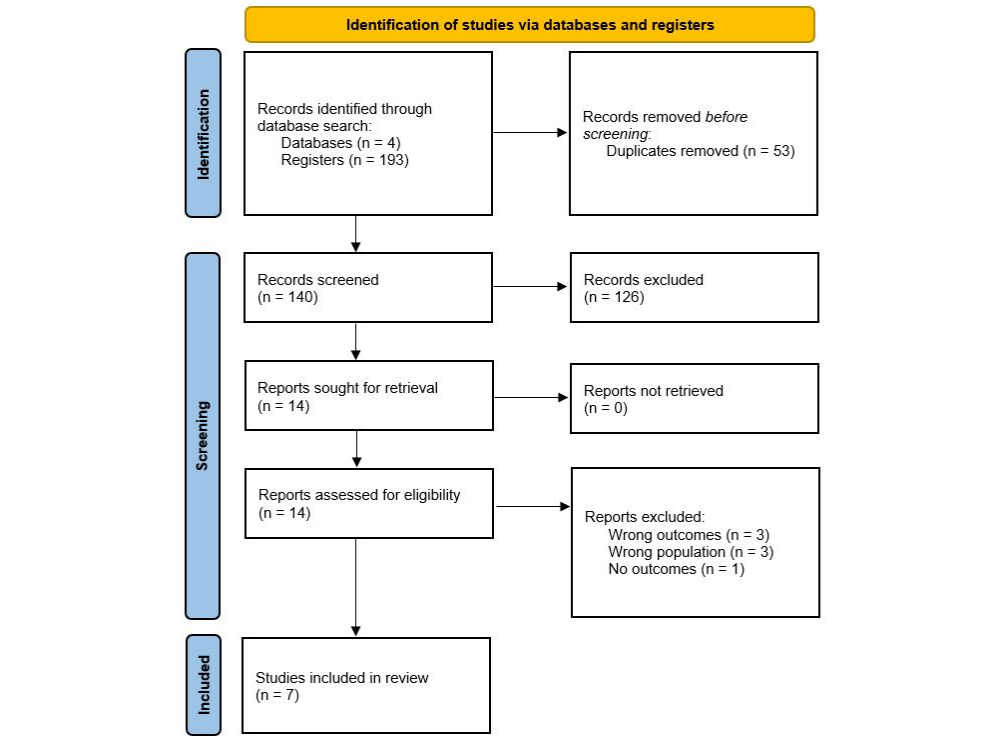
Flow chart of study selection according to PRISMA (Preferred Reporting Items for Systematic Reviews and Meta-Analysis) 2020 guidelines [38].

The initial search identified 193 eligible articles, of which 53 were duplicates. PM and DY screened the remaining 140 abstracts, of which 126 were excluded based on the predefined criteria. In total, 14 papers were chosen for full-text screening, of which 7 studies were excluded, and 7 were included in the analysis. Figure 1 displays the search process and selection of studies in detail.

In addition to author details, relevant characteristics from the selected studies on origin, type of study, sample size, study aim, type and components of intervention, and length of follow-up were extracted and collected in Google Forms. These study characteristics are displayed in Table 1. Outcomes of interest are presented in Table 2.

**Table 1:** Characteristics included studies on digital health interventions aimed at informal family caregivers of people with first-episode psychosis (FEP).

Authors and year	Origin	Type of study and comparison	N values	Study aim	Digital intervention type and components	Length of follow-up
Sin et al, 2014 [51]	United Kingdom	Pilot usability study	20	Test the feasibility, usability, and acceptability of an online intervention, addressing siblings of individuals with FEP.	Web-based. 3 components: information on psychosis; looking after yourself; and Sibling’s blogs and peer forums. All components included fact sheets, interactive modules, CBT^a^-orientated exercises, FAQs^b^, links to further resources, and access to professionals for advice.	5-weeks follow-up.
Kline et al, 2021 [9]	United States	Pilot feasibility Trial	31	Develop and test the feasibility of MILO^c^ for parents and other informal caregivers of people with early psychosis, who are not optimally engaged with treatment.	Video conference. Four 60-minute MILO sessions, teaching the philosophy and basic skills of motivational interviewing, as well as 2 brief practice calls.	12-weeks follow-up.
Kline et al, 2022 [52]	United States	RCT^d^ (waitlist vs IG^e^)	40	Test the impact of MILO on informal caregivers of people with FEP^f^.	Video conference. Four to five 60-minute MILO sessions, in which participants discussed the impact of FEP on the family and caregiver’s own functioning. Further, caregivers learned the foundations and skills of motivational interviewing, and planned, role-played, and reviewed conversations they would like to have with their FEP relative, using their acquired MILO skills.	12-week follow-up (vs waitlist: 6 weeks follow-up).
Gleeson et al, 2023 [53]	Australia	Cluster RCT (CG^g^ vs IG)	164	Determine the effectiveness of altitudes, a web-based digital intervention, on FEP- caregiver stress at 6 months follow-up in a specialist treatment setting.	Web-based. 3 components: online psychoeducation and interactive therapy (divided into specific themes and individual steps); expert moderated social networking; and peer moderation.	6-months follow-up.
Gleeson et al, 2023 [14]	Australia	RCT (CG vs IG)	86	Evaluate whether altitudes improved perceived caregiver stress at 6 months follow-up in a real-world setting in caregivers with a relative with early psychosis, receiving treatment.	Web-based. 3 components: online psychoeducation and interactive therapy (divided into specific themes and individual steps); expert moderated social networking; and peer moderation.	6-months follow-up.
Buck et al, 2023 [54]	United States	Field trial	11	Conduct a user-centered design and testing of a mHealth intervention to support early psychosis caregivers.	mHealth. 4 primary sections: caregiving (psychoeducation and communication coaching through paired lessons and practices); self-care (paired lessons and practices, focusing on managing one’s own stress and well-being); resources (links to external web pages, treatment listings, and videos of others with lived experience, as well as an action plans feature); and tracking (caregivers’ perception of their relative’s symptoms (based on DSM-5^h^ symptoms of psychosis), graphs and indexes of these ratings to track changes over time).	1-week follow-up.
Rus-Calafell et al, 2024 [55]	Germany	Pilot study	25	Evaluate the feasibility and potential efficacy of the first German-moderated online psychoeducation and support program for relatives of people with early psychosis, with the additional purpose of improving accessibility and reducing waiting times for treatment.	Web-based. 5 modules: what is psychosis?; treatment and crisis; communication and emotion; self-compassion; and Health services in Germany. All modules (except module 5) included exercises and downloadable material, as well as games and quizzes to increase participants' engagement. Each module included a module-specific chat to allow for questions and discussion of each module. In addition, in a separate chat (“Time for a break”), caregivers could post general contributions or comments.	12-weeks follow-up.

^a^CBT: cognitive behavioral therapy.

^b^FAQ: frequently asked questions.

^c^MILO: Motivational Interviewing for Loved Ones.

^d^RCT: randomized controlled trial.

^e^IG: intervention group.

^f^FEP: first-episode psychosis.

^g^CG: control group.

^h^DSM-5: Diagnostic and Statistical Manual of Mental Disorders, Fifth Edition.

**Table 2:** Clinical outcomes of interest in included studies.

Authors (year), and type of study		Informal family FEP^a^ caregivers’ user experience	Perceived caregiver stress	Expressed emotion	Parental self-efficacy
**Web-based**					
	Sin et al (2014), pilot usability study [51]	100% (n=17) evaluated the intervention highly and the content as (very) relevant to them (n=16). 59% (n=10) rated the “Ask the Experts” forum positively. Most participants rated the intervention as (very) easy to use. 76% (n=13) would recommend the intervention to other siblings.	—^b^	—	—
	Gleeson et al (2023), RCT^c^ [14]	79% (n=65) of participants reported a positive and constructive experience with the intervention and would recommend it to others (IG^d^).	Lower means in perceived stress at 6 months in CG^e^ and IG, with a statistically significant change for the CG (–1.55 (SD –2.94 to –0.16); *P*=.05) and no significant change for the IG (–1.38, SD –2.85 to 0.10).	Significant improvements in emotional over-involvement and criticism over time (0.22, SD 0.15 to 0.29; *P*=.05), with no statistical difference between CG and IG.	Improvements in parental self-efficacy in CG and IG, with significant changes in the IG (–2.48, SD –4.03 to –0.94; *P*=.01).
	Gleeson et al (2023), cluster RCT [53]	80% (n=12) had a positive and constructive experience with the intervention and 93% (n=14) would recommend it to others (IG).	A significant interaction between group and time on perceived stress (*z*=–2.14; *P*=.03), with higher improvement in PSS^f^ from baseline to 6-month follow-up in the CG.	Improvements in emotional over-involvement (*z*=−2.52; *P*=.01) and dependency (*z*=−2.79; *P*=.01) over time in CG and IG.	Parental self-efficacy was significant in CG and IG, with a greater increase in CG (*z*=2.01; *P*=.04).
	Rus-Calafell et al (2024), pilot study [55]	Participants rated the program positively, describing it as “very helpful”, “important”, and “valuable”. 6 participants stated that the intervention was easy to use, while 5 participants reported technical difficulties.	Statistically significant reductions in the total perceived stress score (t_20_=1.91, *P*=.04) between pre-and post-intervention assessments.	—	Significant positive shifts in caregivers’ beliefs about the degree of control they have over the illness (t_20_=−1.81, *P*=.04). Improvements in perceived self-efficacy (V=136.5, *P*=.48). Caregivers’ coping confidence showed a significant positive increase (V=33; *P*=.007).
**Videoconferences**					
	Kline et al (2021), pilot feasibility trial [9]	89% (n=25) of participants reported that they used the learned skills when communicating with their FEP relative and 26 caregivers said that they would recommend MILO^g^ to a friend in need of similar help.	—	—	—
	Kline et al (2022), RCT [52]	—	Repeated measures ANOVAs indicated significant and large changes in perceived stress (*F*_3,90_=12.41; *P*<.001) in the IG, which remained stable during the 12-week follow-up period.	Repeated measures ANOVAs showed significant and large changes in family conflict (*F*_2.34,70.14_=15.35; *P*<.001), and expressed emotion (*F*_3,90_=13.50; *P*<.001) in the IG. Scores remained stable during the 12-week follow-up period.	Repeated measures ANOVAs indicated significant and large changes in parental self-efficacy (*F*_2.20,59.40_=7.89; *P*<.001) in the IG, which remained stable during the 12-week follow-up period.
**Mobile health**					
	Buck et al (2023), field trial [54]	91% (n=10) of participants reported they would recommend the intervention to others. 82% said they were satisfied with the intervention and 91% found it easy to use. Overall usefulness was rated 8.95 (SD 0.98) out of 10.	Large improvements in overall distress (Cohen *d*=1.77).	Medium-level improvements in expressed emotion (Cohen *d*=.52).	Medium-level improvements in coping self-efficacy (Cohen *d*=.54) and small improvements in coping skills practiced (Cohen *d*=.27).

^a^FEP: first-episode psychosis.

^b^Not applicable.

^c^RCT: randomized controlled trial.

^d^IG: intervention group.

^e^CG: control group.

^f^PSS: perceived stress scale.

^g^MILO: Motivational Interviewing for Loved Ones.

### Study Characteristics

Of the included 7 papers, 6 studies reported on informal family FEP caregivers’ user experience with the digital health intervention [9,14,51,53-55]. A total of 5 studies provided results on perceived caregiver stress and parental self-efficacy [14,52-55], and 4 presented data on expressed emotion [14,52-54]. In total, 4 papers were pilot or field trials [9,51,54,55]. The remaining 3 papers were (cluster) RCTs [14,52,53]. The 2 RCTs by Gleeson et al [14,53] compared enhanced specialized family treatment as usual (STAU) to STAU plus a digital family intervention. As described by Gleeson et al, STAU comprised psychoeducation and support for caregivers in both RCTs. Depending on the service unit, meetings with case managers or psychiatrists and, when indicated, specialist FEP family therapist sessions and access to caregiver support groups were also provided [14,53]. The RCT by Kline et al [52] compared the immediate digital family intervention with a 6-week waitlist control condition.

The majority of studies were conducted in the United States [9,52,54], 2 studies took place in Australia [14,53], and one study each in Germany [55], and the United Kingdom [51]. Across studies, clinical outcomes for a total of 377 informal family caregivers of people with FEP were considered, with the majority of caregivers being female, caring for a male FEP relative in the studies that provided gender details. The majority of informal family caregivers were parents of an ill child, followed by siblings, partners, spouses, and others (for example, custodial grandparents, aunts, or cousins).

The majority of the digital health interventions used in the included studies were web-based (ie, online psychoeducation set up on a website, including online networking and professional moderation [14,51,53,55]), followed by video conferences (ie, psychoeducation delivered through online sessions [9,52]), and one mHealth intervention (ie, a mobile app [54]).

### Principal Findings

#### User Experience

The 6 studies examined informal family FEP caregivers’ user experience based on qualitative surveys [9,14,51,53-55].

Online formats were well accepted and positively evaluated by informal family caregivers of people with FEP [9,14,51,53-55]. Further, digital interventions were perceived as relevant, valuable, and helpful [9,51,55], and most participants found the digital intervention quick and easy to use [51,54,55]. In addition, many participants expressed gratitude for resources specifically targeting informal caregivers of people with FEP [9,51,55].

Taught subjects were rated as (very) useful by participants, with psychoeducational content about psychosis, treatment, and crisis, as well as communication skills being perceived as most important [9,54,55].

Suggestions on how to improve digital health interventions included providing better instructions on how to use all resources embedded in the online application, how to navigate the online platform, and a format suitable for both, computer and smartphone, as well as more time to use the digital intervention [9,51,55]. Further, some participants experienced technical difficulties throughout the study [51,55].

#### Perceived Stress

In total, 5 studies assessed caregivers’ perceived stress levels and reported reductions in stress level scores, regardless of the type of digital intervention. Most studies, except Buck et al [54], used the Perceived Stress Scale (PSS) to measure stress levels [14,52,53,55].

The 2 studies found significant reductions [52,55] in perceived stress level scores pre- to post-intervention, while 3 papers reported improvements in perceived stress [14,53,54]. However, in the two RCTs conducted by Gleeson et al, STAU alone showed higher improvement in PSS scores compared with STAU plus the digital intervention [14,53].

In 3 studies, results remained stable over the follow-up period, ranging from 12 weeks to 6 months [14,52,53]. Gleeson et al [53] found that higher use of the digital intervention was significantly correlated with improvements in perceived stress levels from baseline to six months postintervention.

#### Expressed Emotion

Four studies reported improvements in expressed emotion, including emotional overinvolvement and criticism, and used the Family Questionnaire for assessment [14,19,52,54].

The 2 studies reported significant effects on expressed emotion over time [14,52], while 2 papers reported improvements in expressed emotion before and post intervention [53,54].

#### Parental Self-Efficacy

Results on parental and coping self-efficacy differed between studies and digital interventions used. Further, different questionnaires were used to evaluate parental self-efficacy and coping mechanisms.

Three RCTs found significant improvements in parental self-efficacy [14,52,53], of which one RCT showed a greater increase in parental self-efficacy in the STAU alone group compared with the intervention group [53]. One study reported only medium, nonsignificant improvements in parental and coping self-efficacy between pre- and postintervention assessment [54], while another one found significant improvements in caregivers’ beliefs about illness control and coping confidence [55].

#### Risk of Bias

All 3 RCTs were deemed to have a moderate risk of bias due to blinding, as participants [52] and clinicians [14,52,53] were aware of the group allocation, and because of measurement and selection of reported outcomes. However, data was available for nearly all participants and there were no deviations from the intended intervention or, if amendments were needed, changes were communicated [52]. Detailed results are shown in Multimedia Appendix 3 [56].

For nonrandomized studies, one study received a high risk of bias rating due to possible confounding, selection of participants, and reported outcomes [51]. Further, one study was deemed to have a moderate risk of bias due to the selection of reported results and deviation from intended intervention because of the COVID-19 pandemic [9]. The other 2 studies received a low risk of bias [54,55]. Detailed results can be found in Multimedia Appendix 4 [49].

### Methodological Quality

Based on the QATO, assessing the methodological quality of included studies, most studies received a moderate rating (= one weak rating in one of the 8 assessment categories) and one study got a strong rating (= no weak ratings across the 8 assessment categories [14]). Possible problematic domains included selection bias, study design, and blinding.

Detailed results are shown in Multimedia Appendix 5 [50].

## Discussion

### Principal Findings

This review systematically identified studies exploring the user experience with digital health interventions aimed at informal family caregivers of people with FEP, and their effectiveness on the clinical outcomes of perceived caregiver stress, expressed emotion, and parental self-efficacy.

In line with previous reviews on digital health interventions for informal family caregivers of people with psychosis [39,45], this review found that digital interventions, regardless of their format, were overall well accepted and appreciated by informal family caregivers of individuals with FEP and perceived as helpful and easy to use. Interactions with peers and experts were highly rated [51,55], reflecting a need for education, quicker help, and networking with other people going through the same experience. Most participants reported that they would recommend the intervention to other informal family FEP caregivers, suggesting high satisfaction with the digital format given its accessibility and lower costs associated with the service [9,14,51,53,54]. Further, studies reported positive effects on the clinical outcomes of perceived caregiver stress, expressed emotion, and parental self-efficacy, which are crucial when it comes to supporting the recovery of people with FEP and preventing chronic distress within the family [57-59]. Especially when it comes to perceived stress and expressed emotion, the outcomes of this review are promising, as all studies reported improvements in these outcomes before to after the intervention, which remained stable throughout the follow-up period. However, study results differed in terms of significance, instruments used to measure clinical outcomes of interest and follow-up period. Further, when compared with usual care alone, 2 RCTs found that the digital intervention showed inferior results on perceived caregiver stress and parental self-efficacy compared with the control group [14,53]. Nevertheless, it is important to note that the care available to the control groups in 2 of the RCTs included in this review already comprised gold-standard care, ie, psychoeducation, problem-solving strategies, support, and clinical guidance, which means that while the digital intervention was mainly delivered in a different format, it did not necessarily offer much additional value for participating caregivers [60]. However, this gold standard is the exception in clinical practice, as informal family caregivers often feel excluded from mental health services and left alone when it comes to dealing with FEP, given that not many service units offer such support [21]. Therefore, more studies with well-powered experimental designs are needed, comparing treatment as usual as it is actually common in daily practice (ie, no or inadequate support for informal family caregivers of people with FEP is offered [21]) with digital health interventions to further explore their real impact on clinical outcomes of interest.

Delivering psychoeducation through digital health apps, including networking and skill training opportunities, is currently the most common approach used in supporting informal family caregivers of people with long-term illness through digital health [38]. In line with self-reported key needs of informal family caregivers of people with FEP [24], the interventions identified in this review included education on FEP, effective coping strategies, skill training, and using technology for help. Further, all digital health interventions included in this review used some form of human support (ie, moderated peer forums, access to professional support, or follow-up with participants if the engagement was low). As research has shown that human support has the potential to improve the efficacy of digital mental health apps and engagement with the intervention, this is an important consideration for future studies, for example, when self-guiding tools are tested as opposed to human scaffolding [30,34]. At the same time, the resources required to power human scaffolding should carefully be accounted for when examining the value of digital health interventions in terms of efficiency gains.

Even though results are preliminary, this review suggests that informal family FEP caregivers can profit from the digital format when consuming psychoeducational content as there are many advantages to digital interventions, such as flexibility and easy-to-access resources, the possibility to be part of an online network and feeling less stigmatized, saving time and resources, and instant information exchange [61]. However, subpopulations may prefer different digital formats as some participants in the included studies expressed that they felt negatively affected by experiences their peers shared [55], while others enjoyed reading these posts [51]. This finding suggests that not all types of interventions are suitable for all informal family FEP caregivers. In addition, factors like time spent with the FEP individual should also be considered in future studies, as one of the RCTs showed that this had an influence on parental self-efficacy and expressed emotion [14].

This review also showcases those problems common to digital interventions, including technical difficulties and navigation issues that remained in included studies and are important to address [62]. However, barriers related to accessing support, travel distances, and stigmatization were reduced when using the digital health intervention, something that was positively evaluated by informal family FEP caregivers. Therefore, based on the findings of this review, digital health interventions hold the potential to make caregivers feel more included in mental health services, experience less stigmatization through social networking with peers, and access support and knowledge immediately, which is especially important in FEP, where just-in-time interventions may help to solve a situation of crisis. However, future studies should explore how the format of an intervention may affect its scalability, accessibility, and reach. This is important because different digital interventions will address mental health system burdens and barriers to care in various ways, and robust evidence on these elements will be crucial for informing policy and other decision-makers [34].

There are a few limitations to this systematic review. First, the studies included used different instruments to measure clinical outcomes and follow-up periods ranged from 1 week to 6 months. Further, different types of digital interventions were used and study numbers were too small to compare the different applications. In addition, the majority of papers used different analysis approaches, so a comparison was rather difficult, and therefore, it was not feasible to conduct a meta-analysis. Future studies should use the same instruments and statistical approaches to measure clinical outcomes and determine a similar follow-up period to allow for comparison. Second, the risk of bias and methodological quality was rated as some concerns or moderate for most studies due to possible selection bias (participants and results), no blinding, and the chosen study design. Third, most informal family caregivers were female and parents, which means that the results of this review might not be generalizable to all informal family caregivers of a person with FEP. Future studies should focus on offering evidence-based psychoeducation and support for the whole family system to create a positive family environment and strengthen coping mechanisms, taking individual differences into account [22]. While in past studies on psychosis and caretaking the majority of caretakers have been mothers, research shows that better father involvement in caregiving for children with mental illnesses has a positive impact not only on the person with the mental illness but also on the mothers’ well-being and ability to offer support, given that parents usually represent the biggest support for their child [63,64]. Future digital interventions should therefore recognize the importance of the father role, as well as investigate potential gender differences, given that mothers and fathers tend to have different coping styles when dealing with FEP (ie, mothers tend to use more emotion-focused strategies, while fathers rather turn toward avoidant coping) [65,66]. Considering these differences in future digital health interventions will not only enable greater involvement in the care of both parents but also create better education tailored to individual needs, while supporting each other, particularly mothers, who, based on the current research, often overtake the most responsibility. Furthermore, the majority of included informal family caregivers were white and had a college degree, based on the provided information within the included papers, which is common in studies focusing on digital interventions. Other, often underrepresented groups are more likely to face barriers to digital health such as low quality or no access to the internet at all, little digital health literacy and skills, difficulties taking time off to prioritize mental health, or insufficient resources and accessibility [67]. These barriers are common when implementing digital (health) interventions and future studies need to address these problems and include a more diverse sample. Finally, even though more studies, including much-needed RCTs, were conducted on digital health interventions for informal family caregivers of people with FEP in the last few years, studies on this specific population are still rare and the number of included studies in this review is small. In addition, the RCTs included in this review compared the digital health intervention to gold standard care, which is rather the exception in daily practice and not common in mental health service units. Future RCTs investigating the value of digital health in this area should consider that informal family caregivers often do not receive any or inadequate support when caring for their relative with FEP when choosing the control group [68].

One strength of this review is that well-powered RCTs with a considerable sample size were included. Further, studies not only reported on the user experience with the digital interventions but also provided data on clinically relevant outcomes for informal family FEP caregivers. There were almost no dropouts or missing follow-up data, suggesting a high need and interest of caregivers for such interventions. Patient and caregiver populations were well defined in all included studies, enabling this systematic review to draw conclusions specifically for informal family caregivers of people with FEP. Finally, studies reported also nonsignificant results, helping to reduce publication bias, which is very important when considering future recommendations for the use of digital interventions for informal family FEP caregivers.

### Conclusion

In the past years, there has been a bigger focus on the importance of informal family caregivers in the recovery and illness process of people with FEP, with increased development and use of digital family interventions. The data presented here in both, RCTs and field trials suggest that digital health interventions can contribute to improving perceived caregiver stress, expressed emotion, and parental self-efficacy in informal family FEP caregivers, with participants reporting a positive user experience. While STAU alone partially led to better results in perceived caregiver stress and parental self-efficacy in the RCTs, it is important to note that STAU in the included RCTs already comprised enhanced family care, which is the gold standard of care and not typical in mental health service units. In the studies without a control intervention, all types of digital interventions had a considerable positive impact on informal family caregivers. However, more evidence, especially RCTs comparing routine treatment as it is common in daily practice alone with a digital intervention, is needed to further explore the effectiveness and advantages of digital health interventions and to determine if certain digital formats, for example, mHealth, are more effective and accessible than other types of digital interventions for informal family caregivers of people with FEP. Early family interventions are crucial and digital interventions may offer an effective alternative to in-person appointments, making help and support for informal family FEP caregivers more accessible and reducing waiting times. However, barriers to digital health interventions such as evidence-based implementation in clinical practice, restricted internet, technological problems, and equal access remain, especially in vulnerable groups. Future studies need to address these barriers, while further investigating the effectiveness and scalability of digital interventions to alleviate the burden of care and improve health outcomes and quality of life in informal family caregivers of people with FEP.

## Appendix

Multimedia Appendix 1 PRISMA (Preferred Reporting Items for Systematic Reviews and Meta-Analysis) 2020 checklist.

Multimedia Appendix 2 Sample search strategy conducted in PubMed.

Multimedia Appendix 3 Risk of bias assessment using the Cochrane risk of bias 2 tool for randomized controlled trials.

Multimedia Appendix 4 Risk of bias assessment using the ROBINS-I (Risk Of Bias In Non-randomized Studies - of Interventions) tool for nonrandomized studies.

Multimedia Appendix 5 Methodological quality assessment using the QATO (quality assessment tool for quantitative studies) tool.

## Abbreviations

DUP: duration of untreated psychosis
FEP: first-episode psychosis
MeSH: Medical Subject Headings
PICO: Population Intervention Comparison Outcome
PRISMA: Preferred Reporting Items for Systematic Reviews and Meta-Analysis
PSS: perceived stress scale
QATO: quality assessment tool for quantitative studies
RCT: randomized controlled trial
ROBINS-I: Risk Of Bias In Non-randomized Studies - of Interventions
STAU: specialized treatment as usual
