# Supplementary material for: Digital Health Interventions for Informal Family Caregivers of People With First-Episode Psychosis: Systematic Review on User Experience and Effectiveness
Source: JMIR Ment Health. 2024 Nov 28;11:e63743. doi: 10.2196/63743 (PMC11638689; doi:10.2196/63743)
Supplement: Multimedia Appendix 2 [file mental_v11i1e63743_app2.docx]

Sample search strategy conducted in PubMed.

| **Search** | **Query** | **Results** |
| --- | --- | --- |
| #1 | (Caregiver, Family or Caregiver, Informal or Caregiver, Spouse or Family Caregiver or Informal Caregiver or Spouse Caregiver).mp. | 3830 |
| #2 | Family/ | 85756 |
| #3 | Telemedicine/ | 39478 |
| #4 | Videoconferencing/ | 2367 |
| #5 | Remote Consultation/ | 5834 |
| #6 | Psychotic Disorders/ or Psychoses, Substance-Induced/ | 58180 |
| #7 | "caregiver*".ab,ti. | 96685 |
| #8 | "carer*".ab,ti. | 19542 |
| #9 | "famil*".ab,ti. | 1327426 |
| #10 | psychosis.ab,ti. | 45102 |
| #11 | psychotic disorder.ab,ti. | 3929 |
| #12 | first episode of psychosis.ab,ti. | 5059 |
| #13 | telepsychiatry.ab,ti. | 921 |
| #14 | tele mental health.ab,ti. | 66 |
| #15 | telehealth.ab,ti. | 12479 |
| #16 | "sibling*".ab,ti. | 58113 |
| #17 | "partner*".ab,ti. | 226716 |
| #18 | "friend*".ab,ti. | 138538 |
| #19 | "spouse*".ab,ti. | 20722 |
| #20 | "informal caregiver*".ab,ti. | 4629 |
| #21 | 1 or 2 or 7 or 8 or 9 or 16 or 17 or 18 or 19 or 20 | 1767678 |
| #22 | telemedicine.ab,ti. | 19986 |
| #23 | 3 or 4 or 5 or 13 or 14 or 15 or 22 | 57739 |
| #24 | 6 or 10 or 11 or 12 | 80724 |
| #25 | 21 and 23 and 24 | 30 |
| #26 | "online intervention*".ab,ti. | 1424 |
| #27 | 23 or 26 | 59072 |
| #28 | 21 and 24 and 27 | 40 |
