# Supplementary material for: Digital Health Interventions for Informal Family Caregivers of People With First-Episode Psychosis: Systematic Review on User Experience and Effectiveness
Source: JMIR Ment Health. 2024 Nov 28;11:e63743. doi: 10.2196/63743 (PMC11638689; doi:10.2196/63743)
Supplement: Multimedia Appendix 3 [file mental_v11i1e63743_app3.docx]

Risk of bias assessment using the Cochrane risk of bias 2 tool for RCTs.
