# Supplementary material for: Digital Health Interventions for Informal Family Caregivers of People With First-Episode Psychosis: Systematic Review on User Experience and Effectiveness
Source: JMIR Ment Health. 2024 Nov 28;11:e63743. doi: 10.2196/63743 (PMC11638689; doi:10.2196/63743)
Supplement: Multimedia Appendix 4 [file mental_v11i1e63743_app4.docx]

Risk of bias assessment using the ROBINS-I tool for non-randomized studies.

| Study | Confounding | Participant selection | Classification of intervention | Deviation from intended intervention | Missing data | Measurement of outcomes | Selection of reported results | Overall bias |
| --- | --- | --- | --- | --- | --- | --- | --- | --- |
| Sin et al., 2014 | Moderate | High | Moderate | Moderate | Low | Moderate | Moderate | High |
| Kline et al., 2021 | Moderate | Moderate | Moderate | Moderate | Low | Moderate | Low | Moderate |
| Buck et al., 2023 | Low | Low | Moderate | Low | Low | Moderate | Low | Low |
| Calafell et al., 2024 | Low | Low | Moderate | Low | Low | Moderate | Low | Low |
