# Supplementary material for: Digital Health Interventions for Informal Family Caregivers of People With First-Episode Psychosis: Systematic Review on User Experience and Effectiveness
Source: JMIR Ment Health. 2024 Nov 28;11:e63743. doi: 10.2196/63743 (PMC11638689; doi:10.2196/63743)
Supplement: Multimedia Appendix 5 [file mental_v11i1e63743_app5.docx]

Methodological quality assessment using the QATO tool.

| Study | Selection bias | Study design | Confounders | Blinding | Data collection methods | Withdrawals and dropouts | Intervention integrity | Analyses | Global rating |
| --- | --- | --- | --- | --- | --- | --- | --- | --- | --- |
| Sin et al., 2014 | Weak | Moderate | Strong | Weak | Strong | Moderate | Strong | Strong | Moderate |
| Kline et al., 2021 | Moderate | Moderate | Strong | Weak | Strong | Moderate | Moderate | Strong | Moderate |
| Kline et al., 2022 | Moderate | Moderate | Moderate | Weak | Strong | Moderate | Strong | Strong | Moderate |
| Gleeson et al., 2023 | Moderate | Strong | Strong | Moderate | Strong | Moderate | Strong | Strong | Strong |
| Gleeson et al., 2023 | Moderate | Strong | Moderate | Strong | Strong | Moderate | Strong | Strong | Moderate |
| Buck et al., 2023 | Moderate | Moderate | Strong | Weak | Strong | Strong | Strong | Strong | Moderate |
| Calafell et al., 2024 | Moderate | Moderate | Moderate | Weak | Strong | Moderate | Strong | Strong | Moderate |
